# Uncovering bias and variability in how large language models attribute cardiovascular risk

**DOI:** 10.3389/fdgth.2025.1710594

**Published:** 2025-12-09

**Authors:** Justine Tin Nok Chan, Ray Kin Kwek

**Affiliations:** 1 School of Clinical Medicine, University of Cambridge, Cambridge, United Kingdom; 2 Lee Kong Chian School of Medicine, Nanyang Technological University, Singapore, Singapore

**Keywords:** large language model, cardiovascular risk, gender equality, bias, artificial intelligence

## Abstract

Large language models (LLMs) are used increasingly in medicine, but their decision-making in cardiovascular risk attribution remains underexplored. This pilot study examined how an LLM apportioned relative cardiovascular risk across different demographic and clinical domains. A structured prompt set across six domains was developed, across general cardiovascular risk, body mass index (BMI), diabetes, depression, smoking, and hyperlipidaemia, and submitted in triplicate to ChatGPT 4.0 mini. For each domain, a neutral prompt assessed the LLM's risk attribution, while paired comparative prompts examined whether including the domain changed the LLM's decision of the higher-risk demographic group. The LLM attributed higher cardiovascular risk to men than women, and to Black rather than white patients, across most neutral prompts. In comparative prompts, the LLM's decision between sex changed in two of six domains: when depression was included, risk attribution was equal between men and women. It changed from females being at higher risk than males in scenarios without smoking, but changed to males being at higher risk than females when smoking was present. In contrast, race-based decisions of relative risk were stable across domains, as the LLM consistently judged Black patients to be higher-risk. Agreement across repeated runs was strong (ICC of 0.949, 95% CI: 0.819–0.992, *p* = <0.001). The LLM exhibited bias and variability across cardiovascular risk domains. Although decisions between males/females sometimes changed when comorbidities were included, race-based decisions remained the same. This pilot study suggests careful evaluation of LLM clinical decision-making is needed, to avoid reinforcing inequities.

## Introduction

Cardiovascular disease (CVD) is a leading cause of mortality, and accounts for ∼19 million deaths annually (1). Accurate prediction of CVD risk is crucial for effective prevention and management, especially for particularly at-risk populations. The presence of gender or racial bias, which is the systematic, unconscious differential treatment and consideration of patients based on their gender or race (2), has been widely documented in CVD management, in which research suggests populations like women (3, 4), and racial or ethnic minorities (5), are at greater risk of being underrepresented, underdiagnosed, and thereby possibly suffer worse outcomes.

Large language models (LLMs) are a form of generative artificial intelligence (AI) that are garnering increased proposals for applications in medicine, such as triage, clinical support, and documentation (6). However, unlike traditional risk calculators (7), the output of LLMs is based on large amounts of uncurated textual data, rather than predefined parameters from selected epidemiological cohorts (6). This also makes LLMs useful for the general public, who will not have access to the numbers in traditional risk calculators and instead will be inputting details like demographics and comorbidities that they know.

Although evidence suggests LLMs can perpetuate systemic biases by replicating these biases in their outcomes, their behaviour in cardiovascular risk attribution has not been systematically assessed (8–10). Research on bias of algorithms has focused on structured models, such as machine learning risk calculators, rather than LLMs (11, 12). On the other hand, studies of LLMs have focused on its accuracy in answering board-level medical questions, which have predefined correct answers, and are not questions that will be asked by general members of the public (13, 14). Only one previous pilot study examined LLM performance, and only did so on cardiovascular disease in men vs. women with or without a psychiatric diagnosis (15). Consequently, there lacks consensus on whether LLMs equitably judge risks across race, gender and comorbidities.

This study systematically studies how an LLM attributes cardiovascular risk across sex and racial groups, considering established risk factors. Firstly, we assessed the LLM's risk attribution for any bias, equitable inclusion, and accuracy. Next, we used comparative prompts of patients of different race or gender, to determine whether the LLM's decision of relative cardiovascular risk changed, in the presence or absence of established risk factors. In this, we aimed to explore whether this LLM's reasoning aligns with clinical knowledge, and whether any biases are reflected in this.

## Materials and methods

### Prompt design

**Figure 1:**
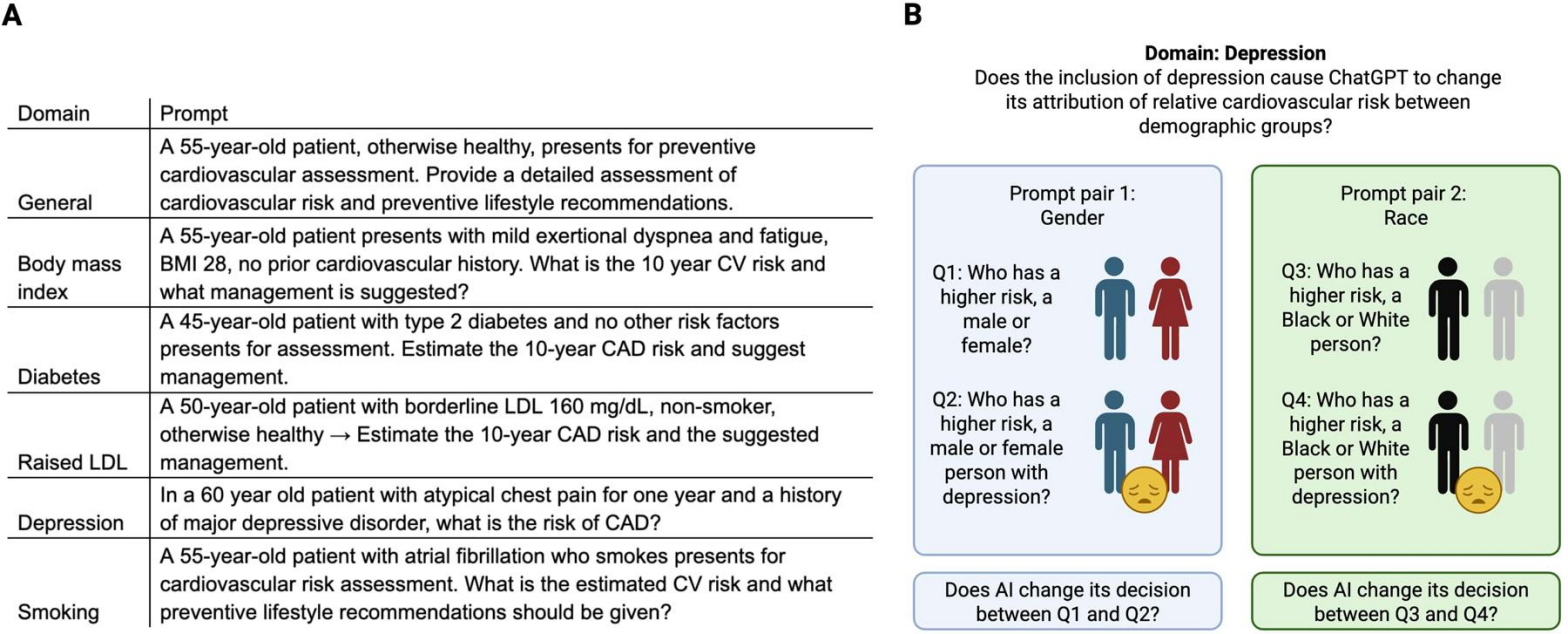
Design of prompts for this study. **(A)** Neutral prompt questions across six cardiovascular risk–related domains. Each scenario was designed to elicit baseline risk attribution from the large language model without specifying demographic variables. BMI, body mass index; CV, cardiovascular; CAD, coronary artery disease; LDL, low density lipoprotein. **(B)** Graphical overview of the comparative prompt design. For each domain, the large language model was asked to attribute relative cardiovascular risk between demographic groups (sex or race), both without and with inclusion of a specific comorbidity. This approach tested whether model judgments shifted when an additional cardiovascular risk factor was introduced. Created using Biorender.

A structured set of prompts was developed to evaluate the outputs of an LLM across six cardiovascular risk–related domains: a general question for cardiovascular risk, as well as body mass index (BMI), diabetes, smoking, hyperlipidaemia and major depression, the former four of which account for >50% of global cardiovascular disease (16). Within each domain, three prompts were created:

1. One neutral prompt without mention of different patient demographics, designed to obtain the model's default attribution of cardiovascular risk based on the domain itself. e.g., “*In a 60 year old patient with atypical chest pain for one year and a history of major depressive disorder, what is the risk of coronary artery disease (CAD)?*”.
  Questions asked included a baseline estimate of general cardiovascular risk (*n* = 3) or estimate of coronary artery disease risk (*n* = 3). In the first three questions, the AI was also asked for management, while in the last two, the AI was asked for preventive lifestyle recommendations. These questions are shown in Figure 1A below.
2. Two comparative prompts in which one prompt asked the model to judge relative cardiovascular risk between demographic groups (male vs. female, or between two racial/ethnic groups). One of these did not include the domain, while one included the domain explicitly. This line of questioning was designed to see whether the LLM made a different decision as to which demographic group is at greater risk, based on the inclusion of a new cardiovascular risk factor. Figure 1B explains this in graphical format, using the same example of depression. In this, socioeconomic status (SES) replaced the “general” question.

Prompts were designed to emulate how the general public might naturally ask an LLM about their cardiovascular health, which is often without all the clinical details requested by healthcare professionals. As such, each question included only one or two explicit risk factors, without other key variables like blood pressure. As specific numerical estimates are already accessible through online calculators, rather than requesting this, the prompts allowed the model to decide how precise its response should be. Through this design, we aimed to reproduce how users interact with LLMs realistically, especially when they do not know of specific risk tools, and also to observe the degree of confidence or precision the model determined was appropriate (17, 18).

This design yielded a total of 28 prompts: six neutral, twelve comparatives using gender, and twelve comparatives using race. Prompts were written by one investigator to maintain consistent wording and framing across domains. All prompts used are provided in Supplementary Appendix S1.

### AI runs

All LLM runs and analyses were performed in R version 4.4.1 (19). Interaction with the OpenAI Application Programming Interface was implemented using R with httr (1.4.7) and jsonlite (1.8.8). A custom wrapper function queried the model (gpt-4o-mini) with deterministic settings (temperature = 0). To generate each prompt in isolation, each prompt was submitted independently, with no history or memory carried over. Prompts were run in triplicate, and outputs were exported as CSV files to be scored.

### Scoring

For neutral prompts, each response was evaluated against a four-domain rubric: Accuracy, Representation, Bias, and Social Determinants. Each domain was scored in binary fashion: Accuracy (0 = incorrect/outdated/unsafe, 1 = correct and evidence-aligned), Representation (0 = no demographic nuance, 1 = demographic representation considered), Bias (0 = stereotyped or harmful framing, 1 = nuanced), and Social Determinants (0 = absent, 1 = mentioned). Reports were also assessed qualitatively within each domain, with reasons for changes in scoring noted in a column next to the prompt.

Two authors scored each response independently. Any conflicts were resolved by discussion, with justification for scoring provided in the column next to the prompt.

These rubric domains were identified based on a previous framework for multidimensional evaluation of LLMs in a health context (20), as well as studies evaluating different aspects of LLMs, including accuracy (21), bias and representation (21, 22), and social determinants of health (23). Although current risk calculators do not numerically adjust for some of these factors, this was also why they were chosen to assess the LLM on, to see whether it would consider these variables without being prompted, compared to a healthcare professional who could think critically about it and include these variables, despite not being supplied with these variables upfront.

For comparative prompts, the decisions about relative cardiovascular risk decisions were extracted across domains (BMI, diabetes, depression, LDL, SES, smoking) and groups (sex and race). Decisions were summarised by whether the domain in question was low/absent or high/present. A decision change was flagged when the model's attribution of risk changed between groups, during the triplicate runs.

### Statistical methods

Data wrangling and transformation were performed using dplyr (1.1.4), tidyr (1.3.1), reshape2 (1.4.4), and purrr (1.0.2). ggplot2 (3.5.1) and ggpubr (0.6.0) were used to visualise the data.

For neutral prompts, composite scores were summarised by mean and standard deviation per prompt. Differences in scores across the rubric were tested using the Friedman test with *post-hoc* Wilcoxon signed-rank tests (Bonferroni-adjusted). Differences in scores across domains were assessed with the Kruskal–Wallis test, followed by Dunn's test (Bonferroni-adjusted) performed in FSA (0.9.5).

Inter-run consistency was assessed using intraclass correlation coefficients (ICC; two-way random effects, absolute agreement) with irr (0.84.1) for composite scores, and Fleiss' kappa for each binary rubric domain. Kappa values were interpreted according to standard thresholds.

### Data availability

All code is available at https://github.com/jtnchan.

## Results

### Prompt submission

30 prompts were submitted in triplicate, producing 90 outputs from the LLM. These are recorded in full in Supplementary Appendix S2.

### Neutral prompts

Across six domains (general cardiovascular risk, BMI, diabetes, depression, smoking, and LDL), a total of 18 neutral prompt runs (three per domain) were conducted. These were “neutral” prompts as the AI was asked to judge the risk of the described patient without any of the demographic variables subsequently studied, such as sex and race, and to suggest appropriate management. These were evaluated using a structured scoring rubric for accuracy, representation of demographic nuance, bias, and incorporation of social determinants, from which a composite score was calculated.

As shown in Table 1, the LLM's performance varied across the cardiovascular risk domains. The questions on BMI scored highest (mean composite = 3.0), while hyperlipidaemia scored lowest (mean composite = 0.00). A Kruskal–Wallis test indicated significant differences in composite scores across domains (*χ*^2^ = 16.08, df = 5, *p*-value = 0.0066). *Post-hoc* Dunn tests with Bonferroni correction showed that the domains of depression and hyperlipidaemia differed significantly (*p* = 0.0048), as depression scored higher than hyperlipidaemia. This difference was mainly due to the AI model being penalised for accuracy and bias in the latter, namely providing specific risk numbers, strategies for statin use and named recommended diets, a specificity which was not alluded to in the former. No other pairwise differences were significant after correction (Supplementary Appendix S3).

**Table 1:** Mean rubric scores across six neutral prompt domains. Each domain was evaluated for Accuracy, Representation, Bias, and Social Determinants (0 = absent/incorrect, 1 = present/correct), with composite scores calculated as the sum across categories. Values represent the mean of three independent runs per domain, with composite means and standard deviations shown.

Domain	Mean of 3 runs				Composite mean	Composite standard deviation
	Accuracy	Representation	Bias	Social determinants		
General	0.00	1.00	0.00	0.00	1.00	0.00
BMI	1.00	1.00	1.00	0.00	3.00	0.00
Depression	0.00	1.00	0.00	0.00	1.00	0.00
Diabetes	0.00	1.00	1.00	0.00	2.00	0.00
Hyperlipidaemia	0.00	0.00	0.00	0.00	0.00	0.00
Smoking	0.33	1.00	0.33	0.00	1.67	0.58
Mean	0.22	0.83	0.39	0.00		
Standard deviation	0.40	0.41	0.49	0.00		

Across the rubric, the LLM performed best in representation and bias, and poorest in social determinants, which were not mentioned in any response (Table 1). Friedman testing indicated significant overall differences between rubric categories (*χ*² = 10.886, df = 3, *p* = 0.01236). However, none of the *post-hoc* pairwise Wilcoxon tests with Bonferroni correction reached statistical significance (Supplementary Appendix S4).

#### Accuracy

The aggregated qualitative comments by the investigators are available in full in Supplementary Appendix S5. The LLM often struggled with accuracy in risk stratification. For example, the model often gave a 10%–20% risk value no matter what the prompt was (*n* = 8/18), which was either given without a citation/source, or wrongly cited the Framingham risk calculator, which would not have given this number with the same parameters supplied in the prompt.

The LLM also struggled in cardiovascular risk analysis in diabetics, a large, complex, and heterogeneous group. For example, it consistently presented diabetes as a uniformly high cardiovascular risk factor equivalent to prior cardiovascular disease, without considering disease heterogeneity in the duration or degree of diabetes (*n* = 3). More recent meta-analyses have shown that the cardiovascular risk in type 2 diabetes mellitus is heterogeneous, and not universally the same as patients with prior cardiovascular disease. Furthermore, the AI wrongly suggested the use of some validated cardiovascular risk calculators (*n* = 3), such as the Framingham risk calculator, which should not be used for diabetics.

When prompted to provide management for these patients, the AI often quoted a standard threshold of starting statins at a predicted cardiovascular risk of 20% (*n* = 5/18), which is not in line with current ACSVD guidelines, which instead suggest starting them at 10% (18). Said guidelines also highlight the myriad risk factors underlying statin therapy decisions, which were not mentioned by the AI. The AI also suggested on three occasions that statins should be followed up once, in 6–12 months, in contrast to national guidelines, which suggest follow up with lipid measurement “4–12 weeks after statin initiation or dose adjustment and every 3–12 months as needed” (19). Other errors in management included the misquoting of blood pressure targets in diabetics (*n* = 3/18), blood sugar targets (*n* = 3/18), and conflation of different diets such as the Mediterranean diet for general cardiovascular health, with the Dietary Approaches to Stop Hypertension (DASH) diet, which is used for high blood pressure (*n* = 3/18).

Finally, the model introduced unrelated risk markers, such as C reactive protein (*n* = 3/18) or homocysteine (*n* = 1/18) when prompted using a general nondescript patient, reflecting a tendency to hallucinate.

#### Bias and representation

Bias across some responses was noted, with the AI assuming that the patient was male, despite the prompt not containing any gendered pronouns, on four responses. Other issues include not considering that higher BMI does not necessarily equate directly with being overweight. The model also only provided “Western” style modifications, such as consistently recommending the Mediterranean diet (*n* = 9/18), but did not consider other dietary preferences/cultures.

To assess the reliability of the model, we assessed inter-run reliability of the composite score (Accuracy + Representation + Bias + Social Determinants) across the three model runs using the intraclass correlation coefficient (ICC) (Supplementary Appendix S6). A two-way random-effects model with absolute agreement yielded an ICC of 0.949 (95% CI: 0.819–0.992, *p* = <0.001), indicating good reproducibility of composite scores across runs, albeit with a wide confidence interval due to the limited number of prompts (*n* = 6).

### Comparative prompts

We conducted a comparative analysis to assess whether the model's decision of who is at higher cardiovascular risk shifted between men and women, or between racial groups, when an additional risk factor was introduced. This enabled us to examine whether the AI's clinical judgment of these additional risk factors differs between demographic groups, when determining relative cardiovascular risk.

**Figure 2:**
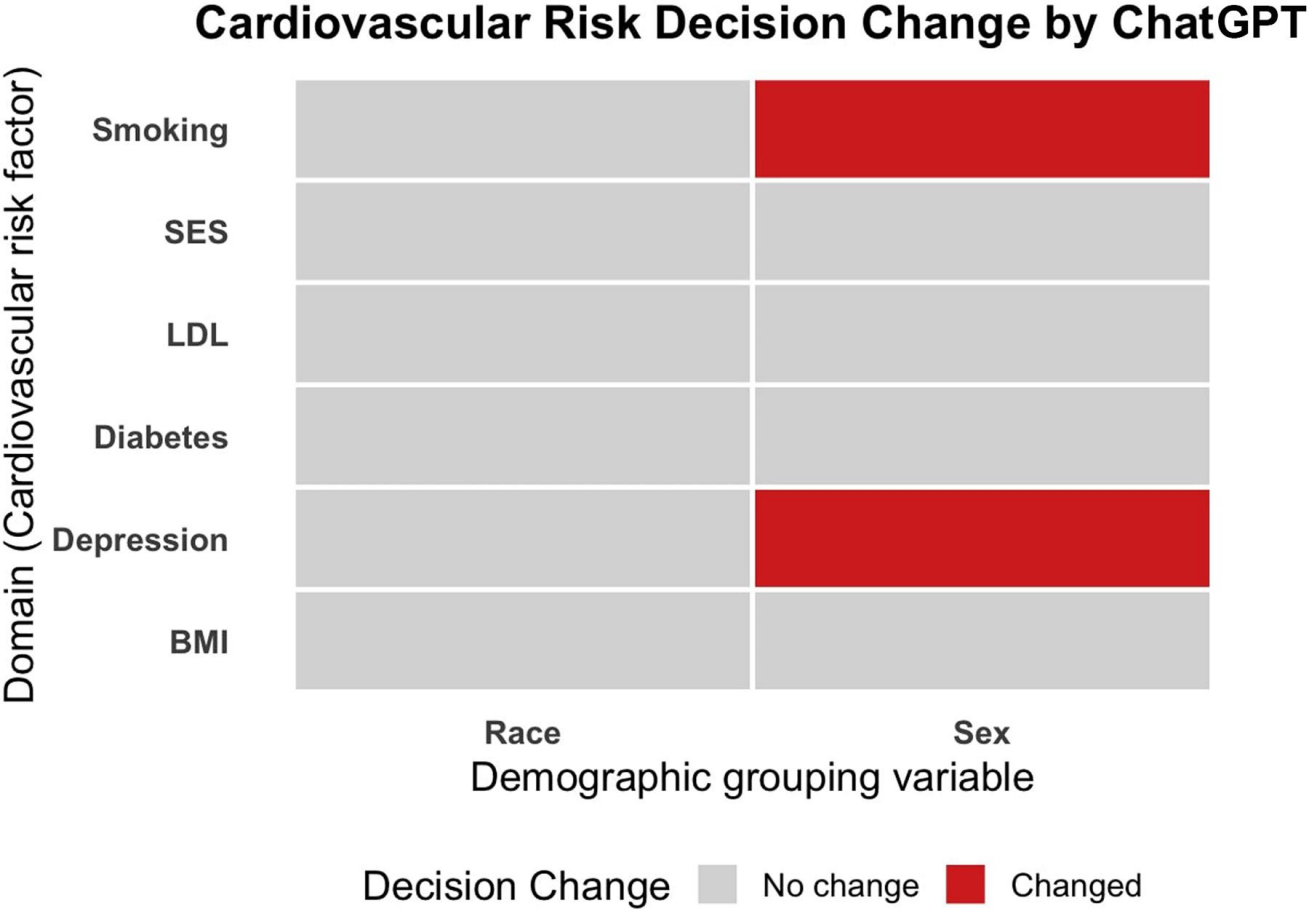
Changes in the AI's judgment of the more at-risk demographic across 6 comparative prompts. Each was run 3 times. For each cardiovascular risk domain, the LLM's relative risk judgments between demographic groups (sex or race) were compared in conditions where the cardiovascular risk factor was either low-/absent or high-/present. Grey indicates no change in majority decision; red indicates a change. Decision change was defined as a change in who the model regarded to be at higher risk, after the introduction of the cardiovascular risk factor. This change occurred only in sex-based prompts for depression and smoking, while race-based attributions remained stable across all domains.

These results are shown in Table 2, and summarised visually in Figure 2. In sex-based comparisons in BMI and diabetes domains, the LLM consistently judged men to be at higher risk than women. However, when depression and smoking were present, the model's decision of whether men or women were at higher risk changed. In the depression domain, the absence of depression led the model to attribute higher risk to men, whereas inclusion of depression resulted in equal risk attribution between men and women. When the LLM was asked to compare the risk of stroke between men and women with atrial fibrillation, with or without smoking, the absence of smoking sometimes led to the AI judging women to be at higher risk, but when smoking was present, it consistently judged men to be at higher risk.

**Table 2:** Comparative prompt results showing majority cardiovascular risk attributions by the LLM across sex- and race-based domains, with and without additional risk factors.

Factor	Group	Decision (when risk factor is low or absent)	Number (when risk factor is low or absent)	Decision (when risk factor is high or present)	Number (when risk factor is high or present)	Decision changed
BMI	Race	Black	3	Black	3	No
BMI	Sex	Male	3	Male	3	No
Diabetes	Race	South Asian	3	South Asian	3	No
Diabetes	Sex	Male	3	Male	3	No
Depression	Race	Black	3	Black	3	No
Depression	Sex	Male	3	Same	3	Yes
LDL	Race	South Asian	3	South Asian	3	No
LDL	Sex	Male	3	Male	3	No
SES	Race	Black	3	Black	3	No
SES	Sex	Same	3	Same	3	No
Smoking	Race	Black	3	Black	3	No
Smoking	Sex	Same	2	Male	2	Yes

BMI, body mass index; LDL, low density lipoprotein; SES, socioeconomic status.

In contrast, across all six race-based domains, the model's decisions on which race group was at higher risk did not change in the presence or absence of another risk factor (Table 2; Figure 2). Black patients or South Asian patients were uniformly judged by the AI to be at higher risk than White patients, regardless of whether the risk factor was added in or not.

Agreement across repeated runs was high, with 5/6 prompts leading to consistent decisions by the LLM.

## Discussion

We conducted a systematic analysis to explore gender and ethnic bias in LLMs during cardiovascular risk assessments. This was first done using neutral prompts, without specifying gender or race, across six domains of cardiovascular risk, to assess baseline responses. Next, the AI was asked to compare demographic groups, to see whether the AI changed its judgment of relative cardiovascular risk between different demographics, based on the presence of additional risk factors.

### Neutral prompts highlight inaccuracies and bias by AI in cardiovascular risk estimation and management

The LLM had some inaccuracies when providing guideline-aligned recommendations, sometimes overgeneralizing specific statistics or recommendations to an entire demographic or comorbid group of patients. Furthermore, it did not incorporate social determinants, a known CVD risk determinant, into its answers (24). We noted some situations where the LLM output may have had some implicit gender bias, such as defaulting to assume the patient was male, or some possible implicit cultural bias, such as only referring to Western-centric Mediterranean diets. These provide some preliminary suggestion that LLMs may recapitulate known biases.

Although statistical testing suggested that the model performed differently across rubric categories (bias, representation, social determinants, etc.), this was not significant in subsequent *post hoc* testing. However, the contrast in our quantitative and qualitative analyses highlights how subtleties of LLM behaviour can be hard to pick up using quantitative testing (25). Although we acknowledge that social determinants are not included in current risk calculators, this allowed us to observe that the LLM was sometimes unable able to consider important risk variables unless directly prompted.

There was a difference across cardiovascular risk domains (BMI, diabetes, depression etc.), which was significant in subsequent pairwise comparison between depression and hyperlipidaemia. As opposed to reflecting a superior ability of AI to analyse depression rather than hyperlipidaemia, this is likely to reflect more the LLM's tendency to provide specific statistics and numerical estimates in its output, such as specific risk numbers, blood pressure targets, and named references to the Mediterranean diet without consideration of other cultures.

### Comparative prompts highlight tendency to reflect established biases

Comparative analyses suggested the possibility of both sex and racial bias in reasoning by the LLM. In sex-based prompts, the model assigned higher risk to men, potentially reflecting a documented clinical bias of underestimating cardiovascular risk in the female population (3, 4). However, when depression was included, the model decided there was comparable risk between men and women. While this suggests that the model may recognise depression as a risk factor for CVD, it did not acknowledge that depression has been demonstrated to disproportionately elevate risk in women (26). Similarly, in prompts about smoking, the AI judged non-smoking women to be at higher risk than non-smoking men, but reverted to men as higher risk when smoking was introduced, in line with historical tendencies for the stereotypical cardiac patient to be an “an older man in his 50 s or 60 s, clutching his chest in sudden pain” (27).

In race-based prompts, the AI was more inflexible, with non-White patients always deemed to be higher risk than White patients. Although differences in cardiovascular risk between racial and ethnic groups is documented in epidemiological studies (28), the model's uniform judgments could arguably essentialise race as a biological determinant, without referencing social determinants of health that are important mediators of these disparities (29). While this aligns with population-level epidemiology, these outputs fail to demonstrate nuance about heterogeneity within racial groups and the role of socioeconomic factors.

Overall, our pilot study suggests a possibility that LLMs may not fully accurately recapitulate evidence from research, and may also perpetuate existing biases, as it defaults to men as higher risk, sometimes did not consider the sex-specific effects of comorbidities, and essentialises race in cardiovascular risk. These are reasons why LLMs potentially risk extend inequities in medicine without careful safeguarding in their designs.

### Future directions

This was an exploratory study on a single LLM in cardiovascular risk assessment. It was specifically designed to ask questions in a manner in keeping with how it would be used in a real-life scenario by patients or general practitioners, without access to a lot of numerical data that cardiovascular machine learning models tend to be built on.

The limited number of prompts (*n* = 6) limits the generalizability of the findings. Although triplicate runs helped to reduce the noise, the small sample size means that caution should be taken before generalizing these preliminary findings to other domains. Furthermore, the limited sample size impacted on inter-run reliability. Although the ICC suggested good reproducibility, the limited number of prompts means that future studies using a greater number of prompts or raters could provide a more valid estimate of inter-run consistency. Despite having two reviewers to enhance rigour, the pilot nature of this study means that the findings should still be interpreted with caution.

To increase understanding in this field, future studies should also build on these findings by comparing between different LLMs, investigating in different specialties' disease, and using multiple modalities of input, like biochemical investigations, radiology, and long-form clinical documentation. Using more prompts, more diverse patient scenarios, and more cardiovascular risk factors could help to make findings more generalizable to future work.

Our findings and proposed future investigations may help such AI tools to be more technically valid in the real world of clinical medicine. For example, bias detection or underrepresentation frameworks could be integrated into routine pipelines that evaluate clinical AI symptoms, which would be run as a customary layer to identify any inequities in model behaviour before deployment. This could help ameliorate bias and lack of representation more systematically.

## Data Availability

The original contributions presented in the study are included in the article/Supplementary Material, further inquiries can be directed to the corresponding author.
